# Unusual Delayed Complication of Pacemaker Leads

**DOI:** 10.7759/cureus.9479

**Published:** 2020-07-30

**Authors:** Anupam K Gupta, Monica I Burgos Claudio, Nir Hus

**Affiliations:** 1 Minimally Invasive Surgery, University of Miami Hospital, Miami, USA; 2 Internal Medicine, Universidad Autonoma de Guadalajara, Guadalajara, MEX; 3 Surgery, Delray Medical Center, Delray Beach, USA; 4 Surgery, Florida Atlantic University, Boca Raton, USA

**Keywords:** pacemaker lead perforation, delayed presentation of perforation, lead dislogment

## Abstract

We present three patients who complained of chest pain secondary to displaced pacemaker leads. They underwent evaluation in the emergency room multiple times for chest pain. Imaging was useful to diagnose misplaced ventricular leads of the pacemaker. The patients needed a pericardial window for the extraction of leads and repair of the heart defect. The perioperative course was managed with a multidisciplinary team of cardiologists and electrophysiologists.

## Introduction

Cardiac pacemakers are devices placed in the chest or abdomen for the management of abnormal heart rhythms [1]. There has been a growing use of pacemakers, and one unusual complication is the displacement of the pacemaker lead [1]. Patients present with a range of symptoms from asymptomatic to life-threatening cardiac tamponade. This article presents the case of three patients who repeatedly presented to the emergency room for chest pain and were found to have misplaced lead eroding the right ventricle to the chest wall and diaphragm. 

## Case presentation

Over the course of one year, we encountered three patients who presented to the emergency room with chest pain (Table 1). All three patients were not pacer-dependent and had a pacemaker placed in the remote past. All three patients had a similar presentation.

**Table 1:** Patient Presentations DM, diabetes mellitus; HTN, hypertension; AV, atrioventricular; HLD, hyperlipidemia; AFib, atrial fibrillation; CXR, chest X-ray; ECHO, echocardiogram.

Age/Sex	Symptoms	Past Medical History	Imaging	Perforation Site
84/F	Chest pain	DM, HTN, HLD, AV block	CXR, CT	Right ventricle
80/F	Chest pain	DM, HTN	CXR, CT	Right ventricle
77/M	Chest pain	DM, HTN, AFib	ECHO, CXR, CT	Right ventricle

Each patient presented with a history of chest pain. The pain was described as intermittent, sharp, and of sudden onset. Additionally, the patients described that the onset of the pain was like a hiccup, but the pain was severe. Each patient had been evaluated multiple times in various emergency rooms, and on each occasion, the patients had stable vitals, physical examination, and blood work. All of the patients had an electrocardiogram and cardiac markers laboratories performed. These tests were considered within normal parameters, and patients were discharged home on antispasmodics or antacids.

**Figure 1:**
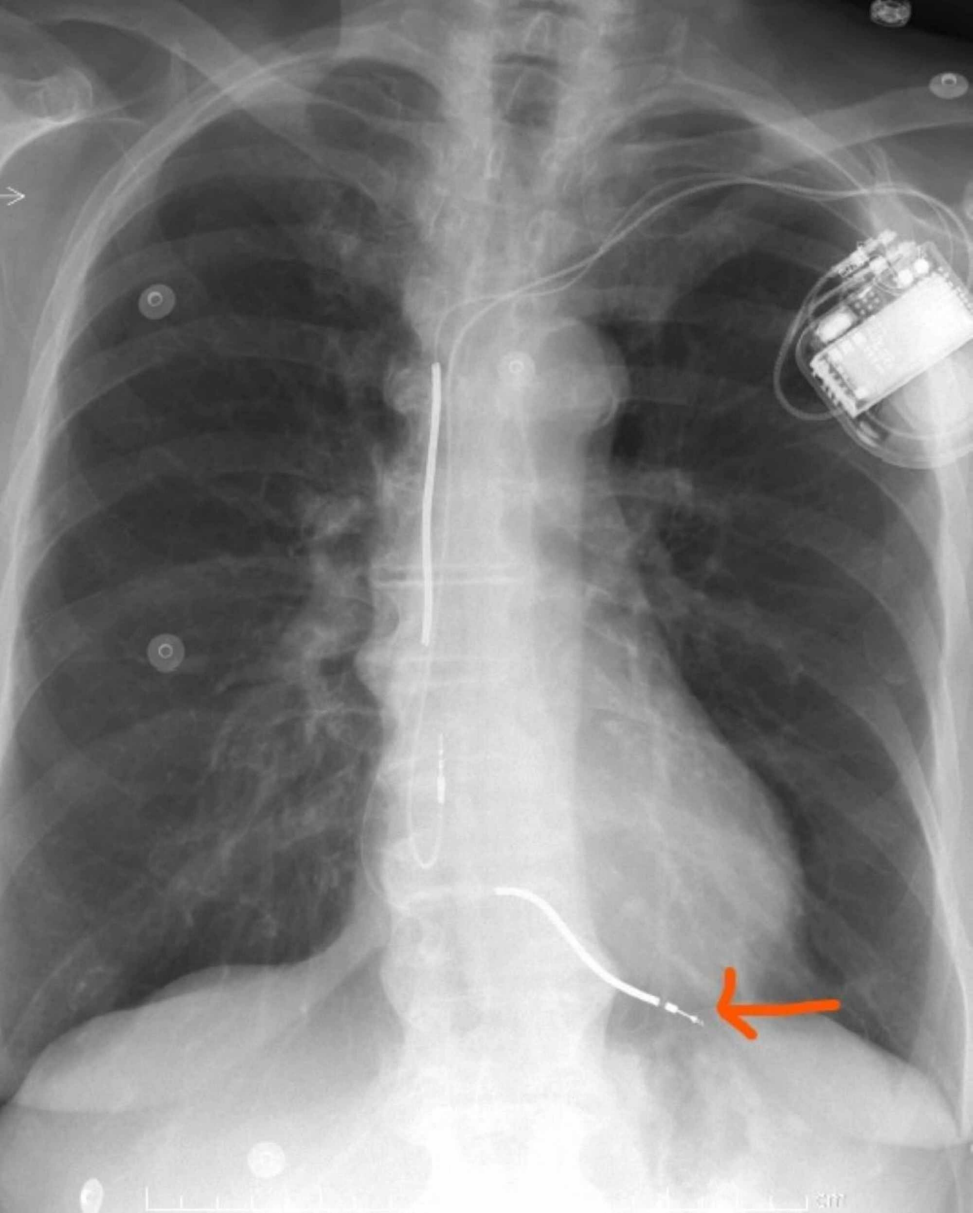
Chest X-ray showing abnormal position of the ventricular lead.

**Figure 2:**
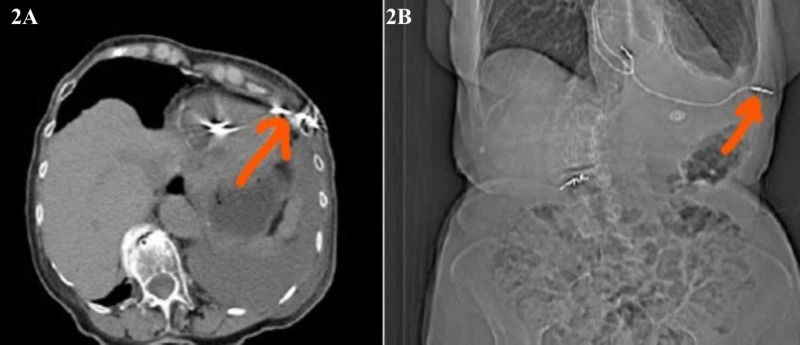
CT scan showing abnormal coronal and sagittal section position of ventricular misplaced lead. (A) CT scan showing coronal section of the misplaced ventricular lead. (B) CT scan showing sagittal section of the misplaced ventricular lead.

The patients underwent imaging testing (chest X-ray, CT, or echocardiography), in which it revealed an abnormal position of the lead (Figures 1, 2). 

The diagnosis of abnormal positioning of the lead was confirmed by CT imaging, and patients were evaluated by a multidisciplinary team. The interdisciplinary team consisted of cardiologists, electrophysiologists, surgeons, and cardiac anesthesiologists. 

**Figure 3:**
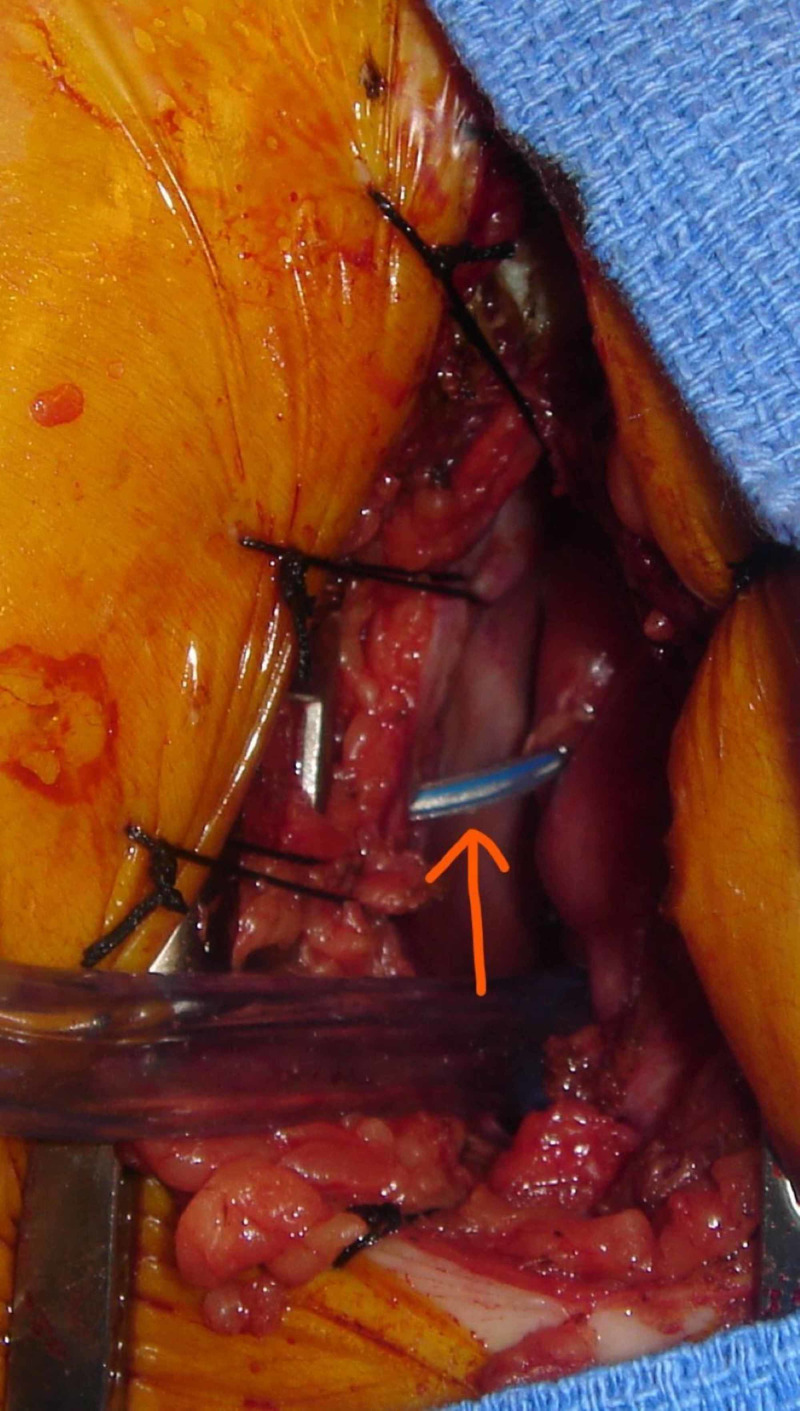
Intraoperative picture showing lead extrusion.

**Figure 4:**
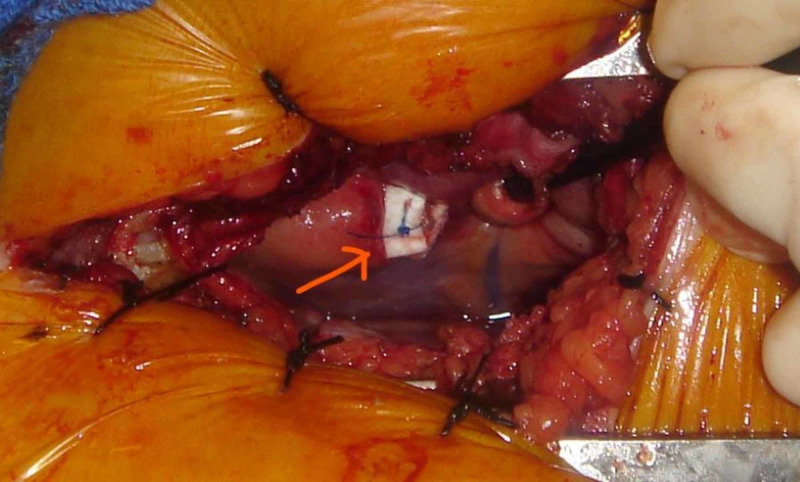
Repair with pledgeted suture after extraction of lead.

An ultrasound was used to identify the pacer leads and identify the intercostal chest space for the site of the incision. Patients were monitored intraoperatively with transesophageal echocardiography. On opening of the pericardium, the pacer leads were identified. Pledgeted sutures were placed around the exit site of leads, and the lead was pulled and cut. The heart defect was closed primarily with preplaced pledgeted sutures (Figures 3, 4). Pericardial and pleural drains were placed and subsequently removed in the immediate postoperative period. Another incision over the pacemaker was used to extract the pacer with proximal half of wires.

## Discussion

A pacemaker is a medical device that provides an electrical impulse to regulate heart rate when the natural cardiac electrical impulse fails [1]. As such, these devices can be either temporary or permanent. The most common indications for permanent pacemaker implantation are sinus node dysfunction and high-grade atrioventricular block. 

A patient is pacemaker-dependent when there is an absence of an intrinsic rhythm of 30 beats/min, if the patient has symptomatic bradycardia or if an underlying rhythm is absent, or more specifically, asystole >5 s without a pacemaker [2]. There are three basic pacemakers, namely the single-chamber, dual-chamber, and biventricular pacemakers [3]. Single-chamber pacemakers have a single lead, placed either in the right atrium or, more often, into the right ventricle. Dual-chamber pacemakers have two leads, one in the right atrium and the other in the right ventricle [4]. As previously stated, none of the patients discussed were pacemaker-dependent. With pacemaker implantation, there is the potential for complications during or after implantation. The rate for the early complications is 4%-5%, and the late complications are at a rate of 2.7% [1]. These complications can be early postoperative, during hospitalization, within 30 days or late (Table 2). 

**Table 2:** Early Complications and Late Complications

Early Complications	Late Complications
Dislodgement of the lead	Decubitus of the pocket and threatening decubitus
Improper location of the lead (left atrium, left ventricle, aorta, pulmonary trunk)	Chronic infection of the pocket unit
Early perforation	Lead-related infective endocarditis
Pocket hematoma	Late lead dysfunction (without exit and entry block)
Pneumothorax	Late perforation/lead erosion
Venous thrombosis	Late recognized old lead dislocation
Early dysfunction of the lead	Lead breaking and lead migration
Early postoperative infection (local and sepsis)	Lead-related venous occlusion

Mechanical factors may cause pacemaker erosion. The incidence of permanent pacemaker perforation is between 0.5% and 2% [5]. A mandatory echocardiographic follow-up evaluation is needed to remain vigilant for this fatal complication.

Acute perforation of the right ventricle or right atrium occurs in 1% of the patients [5,6]. Atrial lead dislodgment is more common than ventricular dislodgements, which occurs due to the thinness of the right atrial wall that has an average thickness of 2 mm, in comparison to the right ventricular wall that is two times as thick [7]. In the ventricle, ventricular apex perforations are more common due to their thinness compared with the interventricular septum. It is hypothesized that excessive tension on the leads predisposes a forward movement through the thinner right atrial or ventricular wall, particularly the apex, causing dislodgment and perforation. Symptoms can range from being asymptomatic to life-threatening pericardial effusions. The most common symptoms include chest pain, dyspnea, syncope, abdominal pain, diaphragm stimulation/hiccups secondary to phrenic nerve stimulation, and left chest muscle twitching, due to stimulation of the left pectoralis major by the lead tip. Cardiac tamponade can develop from a severe pericardial effusion, which can lead to severe hypotension, shock, and cardiac arrest. The most frequently reported predictors of lead perforation are temporary leads, active fixation leads, steroid use, low body mass index, female gender, advanced age, and concomitant anticoagulation therapy [7]. The patients in this discussion include two advanced-age females with low body mass index, and one advanced-age male patient. Identifying lead dislodgement and erosion can be deceiving. An electrocardiogram (ECG) can determine whether the patient is having a permanent or a non-permanent failure, or absence of artifact pacing, which is suggestive of lead displacement [8]. Additionally, an echocardiography, a chest radiography, and a CT scanning can help diagnose perforation and other complications of pacemaker devices. The initial test performed for identifying a displacement is a chest X-ray. Even though a chest X-ray is the preferred test, it can fail to detect lead migration. Regardless of that, it is a valuable diagnostic tool that can detect life-threatening complications associated with cardiac perforation, such as pneumothorax, pericardial effusion, and large-sized hemothorax [9]. A CT scan of the chest, when used synergistically with the X-ray and echocardiography, can visualize the lead tip perforation. 

In the situation where the lead tip is in the mediastinum with no bleeding evidence, a new functional lead can be placed while retaining the previously placed lead [10]. However, this solution can result in the further migration of the malfunctioning lead, which can promote complications later on. Delayed complications of pacemaker leads are unusual. Primarily, management requires a multidisciplinary team. A full multidisciplinary team would include cardiologists, electrophysiologists, surgeons, and cardiac anesthesiologists. Additionally, the approach to pacemaker management should depend on the dynamics of symptoms, pericardial effusion, and hemodynamic status. Closed hemodynamic and echocardiographic monitoring is mandatory during the postprocedural period due to the fact that cardiac tamponade could develop again in a delayed fashion [6].

## Conclusions

Presentation of pacer lead erosion can be variable and non-specific. Imaging is helpful in the diagnosis, should erosion occur. Once diagnosed, patients require surgical removal and further management with a multidisciplinary team. Patients who are not dependent on pacemakers can have a new device implanted at a later date. 
